# The Influence of Social Network Content on the Perception of Smiles—A Randomized Controlled Trial

**DOI:** 10.3390/dj10090168

**Published:** 2022-09-06

**Authors:** Martina Čalušić Šarac, Marko Jakovac

**Affiliations:** 1 Osijek-Baranja County Health Center, 31000 Osijek, Croatia; 2 Department of Fixed Prosthodontics, School of Dental Medicine, University of Zagreb, 10000 Zagreb, Croatia

**Keywords:** social network, smile perception, self-perception of smile

## Abstract

Background: This randomized trial’s objective was to investigate the impact of social network content on the perception of smiles among specialists, doctors, students of dental medicine, and laypeople. Method: A sample of 360 respondents was shown 7 digitally altered photographs of smiles (85.63% female, 14.37% male). Dental specialists, dentists, dental students (first to third year and fourth to sixth year), and laypeople made up the sample. Respondents were asked to rank the images on a scale of 1 to 10, starting with the least appealing and moving up to the most attractive, using a Google Form. Respondents were divided into experimental and control groups at the end of the following month by random selection. The experimental group followed an Instagram profile posting two images of beautiful smiles for seven days, while the control group received no intervention at all. Both groups then completed the same questionnaire again. The comparison of esthetic scores between the experimental and control group was performed using the Mann–Whitney U-test and the difference in test responses between the starting point of the measurement and after exposure to perfect content on social media within individual groups was tested with the Wilcoxon paired-samples test. When comparing the absolute difference of scores, the Mann–Whitney U-test and the Kruskal–Wallis test were used. Results: Respondents in the experimental group rated the rounded embrasures of the incisors with lower esthetic scores compared to the control group that was not exposed to images on the Instagram social network. In those exposed to Instagram (experimental group), laypeople showed significantly greater satisfaction with their own smile after the exposure to Instagram, whereas no such difference was present in the control group. Conclusions: The content of social networks potentially has an influence on smile perception, most visible in the perception of incisal embrasures and self-perception of smile.

## 1. Introduction

Numerous studies have been undertaken about how laypeople and professionals in dental medicine interpret smiles [1,2,3,4,5]. Kokich et al. found that orthodontists, doctors of dental medicine, and laypeople have different perceptions of specific dental esthetic discrepancies [3]. The perception of the tooth shape itself is not the same between the laypeople, i.e., patients, and the population with dental education. The latter prefer a conical-ovoid incisor shape, while patients generally find ovoid incisors the most attractive. Squared incisors are considered the least attractive among both mentioned populations [6]. The shape of the teeth affects the size and shape of the incisal embrasures. They are considered the most pleasing when they are semi-rounded in shape, and this is exactly what is found in conical and ovoid teeth. Squared incisal embrasures are considered un-esthetic, especially among the female population [5]. When discussing the incisal portion of dental crowns, it is important to note that different people perceive the relationship between the length of the central and lateral incisors differently (incisal step). Machando et al. indicated that lateral incisors 1 mm shorter than central were the most desirable among both the dentally educated population and the laypeople, but orthodontists gave significantly lower ratings for photographs in which the incisors are at the same level and in which there is a 2 mm incisal step. Orthodontists are the most sensitive to esthetic defects, probably because their eye has been trained during education. The perceptions of doctors of dental medicine and laypeople did not differ significantly in this study [7]. According to Kokich et al.’s results, a small midline diastema does not impair the patient’s appearance. The authors studied how different groups of respondents perceive the existence of a diastema in the amount of 0.5, 1, 1.5, and 2 mm. The esthetic evaluations of orthodontists were lower when the distance was between 1 and 1.5 mm, while laypeople and doctors of dental medicine marked only a distance of 2 mm as un-esthetic [8]. Several studies show that the perception of dentofacial esthetics is negatively impacted by the presence of a gap between the central incisors [9,10,11,12]. A more recent study, in which the perception of the midline diastema was investigated using videos, also showed that the most attractive smiles are those in which the gaps are not present or are up to 0.5 mm [13]. A pleasant smile is characterized by absolute symmetry of the central incisors. Discrepancies are allowed and tolerated if they are far away from the midline [14].

The use of websites and applications to make and distribute content or to interact in social networking is referred to as “social media” [15]. The development of technology in this era has led to the connection of individuals globally, to easier obtaining and exchange of information through various applications and social networks, but also to the setting of esthetic standards among the younger population [16]. Social networks have become an indispensable part of our lives and their contents can positively or negatively affect us [17]. Instagram is a social network founded in 2010 as an iPhone application, that allows sharing photographs in real-time. The very name of the application comes from the words: “instant” and “telegram” [18], and the basic means of communication through this application is precisely photography. The content of Instagram is full of visual stimuli, either in the form of photographs or in the form of short videos. Although the network was primarily conceived as a tool for entertainment and sharing content with close people, today it is often used for purposes of promotion and advertising for various products. This type of marketing, i.e., web promotion, is considered socially responsible and ecological because it does not require the use of paper and does not create any waste [19]. Instagram offers its users an additional option when sharing content, which is to beautify the content itself by adding various filters, which are built into the application. Thus, before sharing the content, users can beautify it, remove the flaws of the photo they consider inappropriate, or highlight things they want their followers to notice. In the field of dental medicine, there is not enough published research on the topic of the influence of social media content on smile perception or self-perception, although many dental clinics use them to bring their work and achievements closer to patients by posting photos or videos [20]. The female population also uses content from social networks, more precisely from Instagram, when choosing a dental clinic [21]. A study conducted among students divided into an experimental group, which looked at images of beautiful smiles on Instagram, and a control group, which looked at pictures of nature, showed that looking at images of beautiful smiles reduces self-satisfaction in a short period of time [17]. Studies involving television content have shown that watching short commercials can also change women’s perception of their own appearance [22]. Since images of famous people with ideal body proportions are mercilessly posted on social networks (and these profiles attract followers since they are mostly public), the number of publications discussing the influence of social network content on how people perceive the body and face is constantly rising [23]. Plastic surgeons in this pandemic and post-pandemic period had an increased number of requests for various corrections, among other things, the removal of impurities on the skin of the face because the images they see on Instagram and other social networks are passed through filters that make the skin smooth and shiny [24]. It is important to know how much influence photographs and videos that we encounter every day on social networks have on the perception and self-perception of a smile. The purpose of this randomized research was to ascertain whether social network content affects how specialists, dentists, dental students, and laypeople perceive smiles. The following hypothesis was tested: the perception of specialists and doctors of dental medicine is not influenced by the content of social networks, while their content will influence the perception of laypeople.

## 2. Materials and Methods

### 2.1. Participants

On social media, participants were recruited in January and February of 2022. The Sampson et al. study, which calculated the sample size using a mean effect size of 0.5 to determine the significant difference in facial satisfaction before and after exposure to social network scores between experimental and control groups with 80% power using a two-sided test, was used as guidance on sample size, because there are not many studies of this kind. They found that a sample of 128 participants would be adequate [17]. The total number of respondents in this research was 360. The sample included dental specialists, dentists, dental students (1st to 3rd year and 4th to 6th year), and laypeople. The group of dental specialists consisted of orthodontists, prosthodontists, and periodontologists. They were divided using the option “random between” in the Microsoft Excel program into control and experimental groups (Table 1). A statistically significant difference was found in the age of the subjects between the control and experimental groups (*p* < 0.001).

Dental technicians and dental hygienists were excluded from the laypeople group because they have some degree of professional dentistry knowledge. 

### 2.2. Method

**Figure 1:**
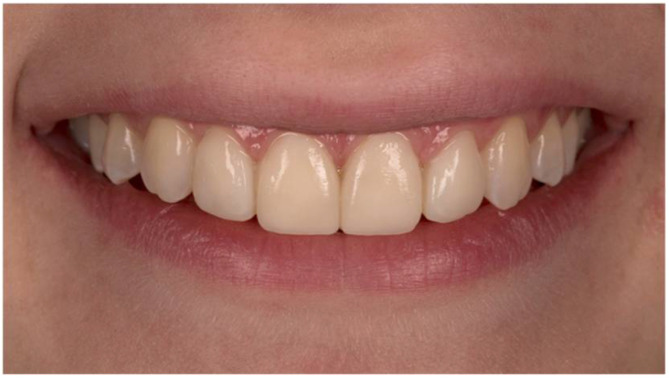
Original image with semi-rounded incisal embrasures, an incisal step of 2 mm, and no diastemas or “black triangles”.

**Figure 2:**
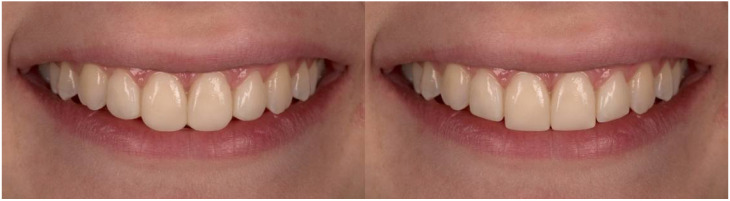
Rounded and squared incisal embrasures.

**Figure 3:**
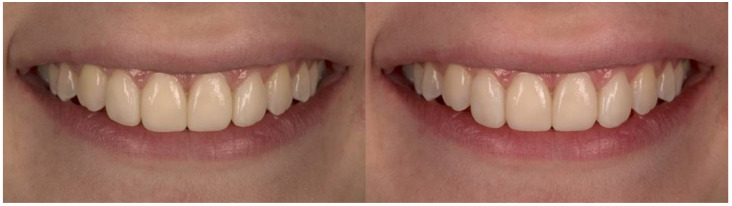
Incisal step of 1 and 0 mm.

**Figure 4:**
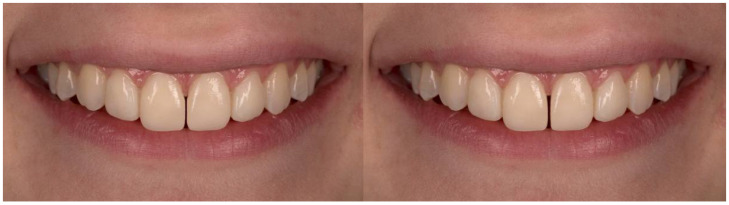
Midline diastema of 0.5 and 1 mm.

A macro-lens of 105 mm and f/2.8 was used to capture the frontal view of a woman’s smile on a Nikon D750 camera. The images were then imported into Adobe Photoshop Lightroom 6 (version 6.0, San Jose, CA, USA), where the parameters for the study were changed. A millimeter scale was used to translate the measurement of the mesiodistal width of the central right incisor to the images. The semi-rounded incisal embrasures, the 2 mm incisal step between the central and lateral incisors, and the absence of diastemas and “black triangles” between the teeth are all visible in the original shot (Figure 1). Squared and rounded incisal embrasures were two alterations made to the incisal embrasures (Figure 2). There were two changes made to the incisal step: a 1 mm incisal step between the central and lateral incisors, and no incisal step at all (Figure 3). A midline diastema of 0.5 and 1 mm was made (Figure 4).

A total of seven images were made, including the original one.

**Figure 5:**
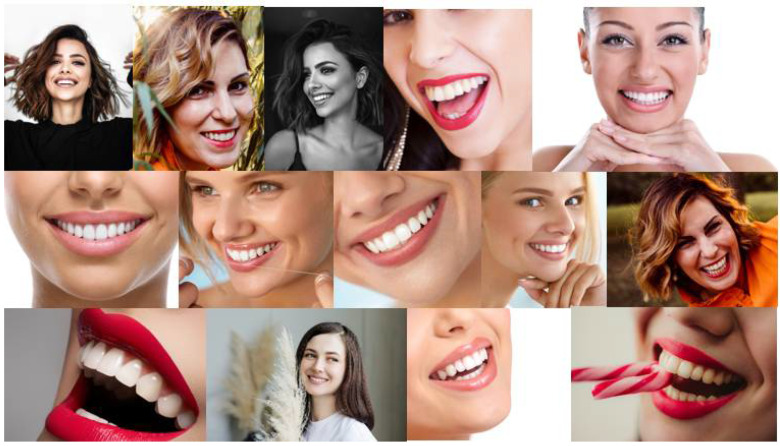
Photographs posted on Instagram.

The comparability of survey results between various media was evaluated prior to choosing the best testing method. Twenty-four respondents evaluated the identical images seven days apart, first on a computer, then on a mobile device, and lastly on a sheet of paper. Due to a high lack of statistically significant differences between responses provided by different media (*p* = 0.63, repeated-measures ANOVA) and a high degree of agreement (intraclass correlation coefficient (ICC) of 0.864, with a 95% confidence range from 0.709 to 0.944) with the average measure, it was decided to perform the research using mobile devices or computers. A Google Form (questionnaire) was created, which in the first part collected the socioeconomic and demographic data of the respondents, and the second part showed randomly ordered images that respondents rated from 1 (least attractive) to 10 (very attractive). The respondents had to rate satisfaction with their own smile on the same rating scale (from 1 to 10). One month after completing the questionnaire, half of the respondents were selected via the “random between” option in the Microsoft Excel program and asked to follow a profile on the social network Instagram, created for the purpose of conducting the research. For a period of 7 days, through the “Story” option on Instagram (so that the examiner would have an insight into whether the subjects viewed the targeted content), they looked at images of smiles, which meet all the criteria of a beautiful smile, with an emphasis on the parameters that are examined in this research. Before selecting the images that were posted on Instagram, 5 experienced clinicians, 2 of whom were specialists in orthodontics, 2 specialists in dental prosthetics, and 1 specialist in periodontics, evaluated 65 images of smiles, and the 14 images that received the highest score were posted on Instagram (Figure 5). An incisal step of 2 mm was present in the majority of smiles (11), while 3 smiles showed an incisal step in the amount of 1 mm. Semi-rounded incisal embrasures were present in the 10 photographs, 3 photographs showed squared embrasures, and only 1 showed rounded incisal embrasures.

The database of 65 photos was compiled from personal photographs of the researchers and images that are available for download on the Internet. The “Story” option broadcasts the image for a maximum of 15 s, and it disappears from the profile after 24 h. The subjects were looking at two images a day, at least once. After the expiration of 24 h, all images that were posted via the “Story” option were saved in “Highlights”, so that respondents had the opportunity to view them even after the end of the broadcast. On the eighth day from the start of broadcasting the images on Instagram, the respondents were sent the same questionnaire through the Google Forms service. The control group (the other half of the respondents) also completed the same survey a second time at the same time. 

### 2.3. Intra-Rater Reliability

Five randomly selected respondents from each occupational group, who were not exposed to dental content on social networks, evaluated two identical photographs of a smile (Figure 1), with a gap of two weeks. Given the non-normal distribution of the data, the Wilcoxon paired-samples test was used. There was no significant difference in image scores before and after 2 weeks (*p* (all groups) > 0.05), with a Cohen’s kappa coefficient for all groups of κ > 0.45 (moderate agreement between 2 measurements).

### 2.4. Statistical Analysis

The data were organized in Excel tables and processed in a statistical program (TIBCO Statistica™ 14.0.0.15). The comparison of esthetic scores between the experimental and control groups was performed at the initial point of measurement and at the point of measurement 30 days after exposure to dental content on the social network between the 2 main groups (control vs. experimental group), and for each of the groups separately (laypeople/1st–3rd year students/4th–6th year students/doctors of dental medicine/specialists in the control vs. laypeople/1st–3rd year students/4th–6th year students/dental medicine doctors/specialists in the experimental group) with the help of the Mann–Whitney U-test. The differences in test responses between the starting point of the measurement and after exposure to perfect content on social media within individual groups were tested with the Wilcoxon paired-samples test. The absolute difference between pre- and post-exposure scores was calculated. The Mann–Whitney U-test was used to compare the differences between experimental and control groups, and the Kruskal–Wallis test was used to compare the differences between the specialists, dentists, students, and laypeople in the experimental and control groups.

## 3. Results

At the starting point of the measurement, there was no statistically significant difference in the assessment of one’s own smile, nor in the assessment of individual anomalies between the experimental and control groups (*p* > 0.05). Mean scores, standard deviations, medians, and confidence intervals for each individual anomaly in the experimental and control group, in the first testing, are shown in Table 2. The differences between the experimental and control groups by individual occupations in the first survey are shown in Table 3.

Mean scores, standard deviations, medians, and confidence intervals for each individual anomaly in the experimental and control groups at the second measurement point are shown in Table 4, while Table 5 shows the differences between the experimental and control groups by individual occupations after exposure to the social network.

**Figure 6:**
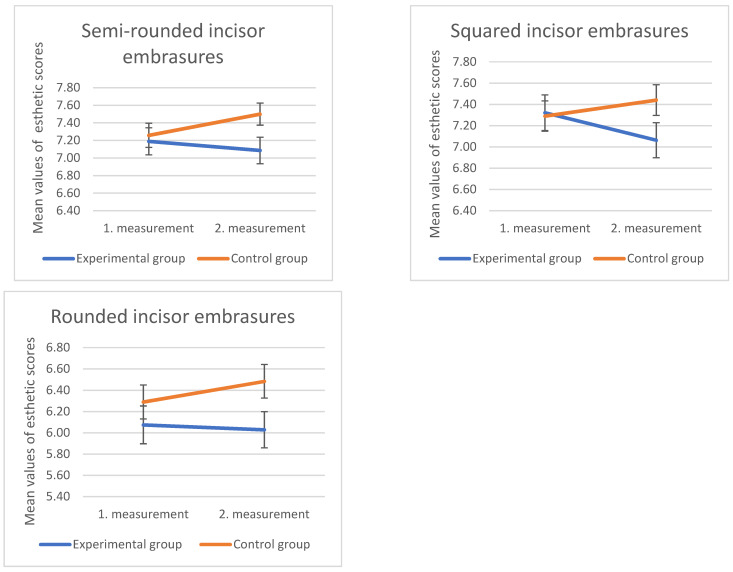
Mean values and standard errors (error bars) of the ratings of the control and experimental groups for photographs showing semi-rounded, squared, and rounded incisor embrasures, in the first and second measurements.

A significant difference in the esthetic scores of the control and experimental groups was found for the photograph showing rounded incisor embrasures (U = 14,129.50, Z = −2.08, *p* = 0.03) (Figure 6). Respondents in the experimental group rated the rounded embrasures of the incisors with lower esthetic scores compared to the control group that was not exposed to images on the Instagram social network (control group 6.48 ± 2.15, experimental group 6.03 ± 2.24). The group of “Laypeople” who were exposed to images on Instagram rated the squared incisor embrasures significantly lower compared to the same group of subjects from the control group (experimental group = 7.53 ± 2.03, control group = 8.21 ± 1.64). No statistically significant difference in the esthetic scores of the control and experimental groups was found for images showing semi-rounded incisor embrasures (*p* > 0.05).

**Figure 7:**
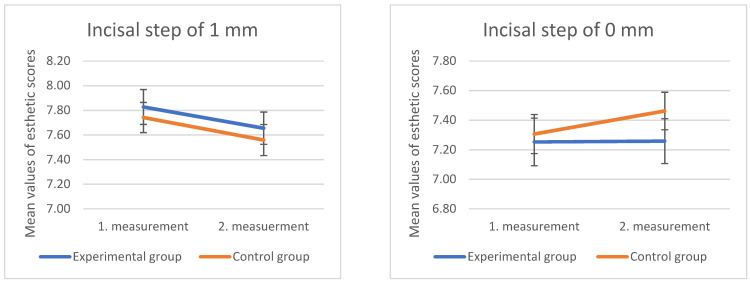
Mean values and standard errors (error bars) of the ratings of the control and experimental groups for images showing lateral incisors 1 mm shorter than central, and lateral incisors at the same level as central, in the first and second measurements.

No statistically significant difference was found in the ratings of photographs showing 0 and 1 mm of incisal step between the control and experimental groups in the second measurement. Figure 7 shows how the scores for the image showing central and lateral incisors of the same length were lower in the second survey within the test group, and higher in the control group at the same time point.

**Figure 8:**
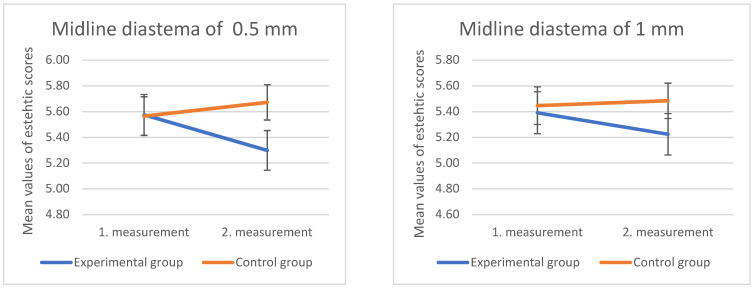
Mean values and standard errors (error bars) of the ratings of the control and experimental groups for images showing a midline diastema of 1 and 3 mm, in the first and second measurements.

No statistically significant difference was found in the second survey in the ratings of images showing a midline diastema of 0.5 and 1 mm (*p* > 0.05). Figure 8 shows how the esthetic scores for both images showing midline diastema were lower in the second survey within the test group, and higher in the control group.

**Figure 9:**
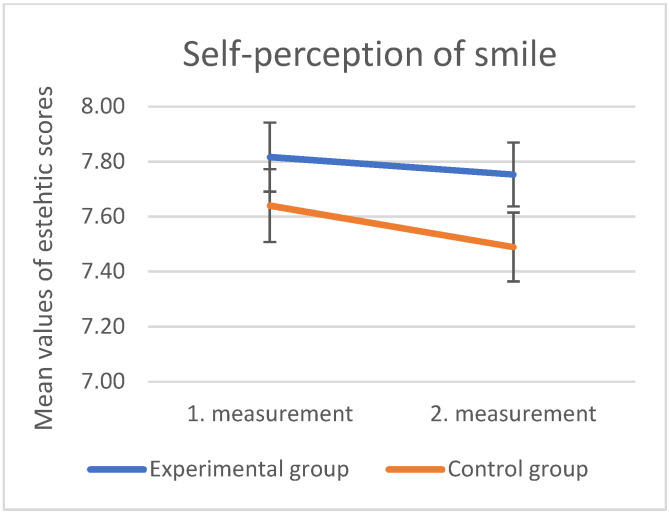
Mean values and standard errors (error bars) of the scores of the control and experimental groups for the parameter “self-perception of smile” in the first and second measurements.

Self-perception of smile did not differ significantly between control and experimental groups after exposure to social media (Figure 9). In those exposed to Instagram (experimental group), laypeople showed significantly greater satisfaction with their own smile after the exposure to Instagram, whereas no such difference was present in the control group (mean values of the scores of the “laypeople” experimental group pre-exposure = 7.63 ± 1.87, post-exposure = 7.62 ± 1.77) (*p* = 0.03).

Statistics performed on absolute changes of scores showed that there was no significant difference between the experimental and control groups. However, when observing only dentists, differences were found between the control and experimental groups for the image showing a diastema of 1 mm (*p* < 0.05). Additionally, when observing students from 1st to 3rd year, there was a significant difference between the control and experimental groups for image showing a diastema of 0.5 mm (*p* < 0.05). Within the experimental group, no significant differences were found between occupational groups, after exposure to content from the social network. A significant difference was found within the control group, between dentists and laypersons, for the parameter that examines the self-perception of a smile (*p* = 0.02), and between students from 4th to 6th year and laypersons for the image showing a midline diastema of 0.5 mm (*p* = 0.004).

## 4. Discussion

The human face is a portion of the body that is frequently exposed and becomes apparent during interpersonal contact. Even without spoken communication, it gives us a lot of information, such as gender, age, identity, interests, feelings, attractiveness, or origin [2]. The perception of attractiveness varies from person to person and determining what is beautiful is mostly a subjective process. Through numerical symmetries and proportions, philosophers and scientists have attempted to measure beauty throughout history by dividing the face into quadrants or thirds [25,26]. In the last decade, we have witnessed an increased interest in research on facial attractiveness. The development of computer graphics has made it possible to objectively investigate the characteristics that are considered attractive within a certain population. Averageness, facial symmetry, and sex-specific traits are associated with attractiveness [27]. All the mentioned parameters can be changed, emphasized, or completely removed on the same face with the help of computer programs to test their acceptability in society. Such programs allow us to change only one parameter on the same model, while leaving everything else the same. In this way, we isolated the desired characteristic and reduced the distraction of the respondents with other features present on the face of our model. Before the development of such computer programs, research of this type was possible only on photographs of individuals who really possess the characteristics we wanted to investigate, but such comparisons were then difficult to carry out, because the examinee’s attention was focused on the complete experience of the researched person, and not on one characteristic which appears in several gradations on the same face. This research was conducted with the help of one female model. To reduce the distraction of respondents, all investigated parameters of the smile were modified on the image of the same smile. This research investigated how social networks affect perception and self-perception of smiles. The total sample of respondents was divided into a control and an experimental group. The experimental group followed an Instagram profile where photos of a beautiful smile were published twice a day, for seven days. After that, within 48 h, they filled out the same survey as at the beginning of the study. The control group, who did not follow the contents of the Instagram profile, filled out an identical survey at the same time. Looking at the entire sample of the experimental and control groups, a significant difference in ratings existed only for the image showing rounded incisal embrasures. The experimental group rated the image with significantly lower esthetic scores than the control group. It is not entirely clear why the content published on Instagram had such an impact on the perception of incisors, but if we look at the images the experimental group was looking at, the semi-rounded incisal embrasures are dominantly present. Looking at the results by occupational groups, it is evident that the “laypeople” in the experimental group rated the image showing squared incisal incisions significantly lower than the “laypeople” within the control group. Even in the first survey, the ratings of the “laypeople” were highest for squared incisal embrasures and were significantly higher than the ratings of the photograph showing semi-rounded incisal embrasures. The images posted on Instagram also influenced the “laypeople” whose ratings were significantly lower in the experimental group, which can also be attributed to the choice of images posted on Instagram. 

In addition to a substantial difference in how incisal embrasures were perceived, only the “laypeople” group showed a significant difference in how they saw their own smiles. Laypeople exposed to Instagram (experimental group) showed significantly greater satisfaction with their own smile after the exposure to Instagram, whereas no such difference was present in the control group. Looking at the entire sample of the experimental and control group, the self-perception of smiles did not significantly differ. Social networks had the most significant influence on the self-perception of “laypeople”. Papers about the influence of television commercials and fashion magazines on the perception of face, smile, and body esthetics have been published [22,28,29]. Brief viewing of advertising content can change self-perception, especially in women [30]. The research conducted by Laus et al. showed that advertising content, which shows a beautiful and pleasant smile, does not cause a big change in one’s own perception of the smile. Their subjects were divided into two groups: the experimental group, who watched advertising content consisting of esthetically attractive smiles, without visible malocclusions, for 1.45 min, and the control group, who watched advertising content that did not include people for the same duration [22]. The duration of the advertising content was short and broadcast only once, so the impact on self-perception was not proven. In this study, the visual stimulus lasted longer. The experimental group was looking at photos of a beautiful smile for seven days. Two photos were broadcast every day, one in the morning, and one in the afternoon. Contrary to expectations, “laypeople” were less critical of their own smile after watching the content than in the first survey, a month before following the content on the Instagram profile. It can be assumed that the laypeople, after being exposed to the photos in the first test, were sensitized and looked more and thought about their own smile. For some individuals, observing smiles in the first survey may have prompted them to visit dentists to improve their appearance. People who focus on positive and potentially positive changes in themselves generally show a higher degree of self-confidence, which may have been stimulated in the “laypeople” of the experimental group [31]. Our results are consistent with the research of Pop et al., who showed that the use of the “Snapchat” social network leads to an increase in self-satisfaction among its users [32]. However, research that shows how the content of social networks negatively affects self-perception has also been published [33,34]. Most of the published research deals with the impact of the content of social networks on the self-perception of the body, that is, how much photos of slim bodies negatively affect the population, especially women. More recently, researchers are examining the impact of the “body positivity” movement on self-confidence [35], but the impact of photographs of dental content remains unclear. Sampson et al. conducted a randomized controlled trial, in which the control group was exposed to photographs of nature, while the experimental group viewed photographs of idealized smiles. After the exposure, the experimental group showed significantly lower satisfaction with their own appearance [17]. On the contrary, the results of our research showed that photos of beautiful smiles had a positive effect on laypeople, who were more satisfied with their smiles after viewing the photos on Instagram. Almost all images posted on Instagram included smiling people, so maybe that part of the positive energy was transferred to the “laypeople”. The other groups (dental students/dentists/dental specialists) did not change their opinion about their own smile, which is understandable, given that all these groups have a certain amount of dental education, so they encounter beautiful smiles much more often than “laypeople”. 

When observing the absolute change of scores, a significant difference was found between occupational groups in the control group: the self-perception of smiles differed between dentists and laypeople, but this difference was not visible in the experimental group. The images posted on Instagram may have approximated the perception of these two groups. 

This study examined the short-term impact of social networks on self-perception and smile perception, as respondents filled out the survey a second time immediately after being exposed to Instagram content; however, the perception of one’s own smile is subject to external influences, which cannot be completely excluded. The distribution of dental specialists in the control and experimental groups was not uniform, probably because fewer dental specialists use the Instagram social network, and this could have an impact on the results. Another limitation of the research is that the long-term influence of social networks was not examined. The long-term impact of social networks should be examined in a separate study, in which emphasis would be placed on the long-term impact in isolation, to avoid the lack of a controlled environment when filling out the questionnaire. There are more parameters that define the beauty of a smile, such as midline position, incisal inclination, smile line, buccal corridors, amount of visible gingiva, etc., that were not examined in this study. These characteristics should be examined in future studies. 

## 5. Conclusions

The content of social networks potentially has a short-term influence on smile perception, most visible in the perception of incisal embrasures and self-perception of smile. Rounded incisor embrasures were rated with lower esthetic scores after viewing images of a smile showing semi-rounded incisor embrasures on Instagram. Laypeople, who looked at the same images, rated squared incisor embrasures significantly lower, compared to laypeople who did not follow the Instagram posts between the two tests. Images of beautiful smiles posted on Instagram had a positive effect on the self-perception of smile among laypeople. After following the content on Instagram, they were more satisfied with the appearance of their own smile.

## Figures and Tables

**Table 1:** Demographic and socioeconomic data of the participants.

	Experimental Group (*n* = 174)	Control Group (*n* = 186)
**Age**	30.07 ± 7.16 *	34.40 ± 8.57 *
**Gender (%)**		
*Male*	14.37	29.03
*Female*	85.63	70.97
**Education (%)**		
*Laypeople*	48.27	46.24
*Dental students (1st to 3rd year)*	14.94	6.45
*Dental students (4th to 6th year)*	15.53	7.53
*Dentists*	16.09	22.58
*Dental specialists*	5.17	17.20
**Filled out the questionnaire on (%)**		
*Smartphone*	94.25	95.7
*Personal computer*	5.75	4.30

*n*: Number of participants, * *p* < 0.05

**Table 2:** Mean scores, standard deviations, medians, and confidence intervals for each individual anomaly in the experimental and control group, in the first testing.

	Experimental Group (*n* = 174)					Control Group (*n* = 186)				
	Median	IQR	Mean	SD	(95% CI)	Median	IQR	Mean	SD	(95% CI)
**Self-perception of smile**	8.00	2	7.82	1.66	1.50–1.85	8.00	2	7.64	1.81	1.64–2.01
**Semi-rounded incisor embrasures**	8.00	3	7.19	2.03	1.84–2.27	7.00	3	7.26	1.87	1.70–2.08
**Squared incisor embrasures**	8.00	3	7.32	2.20	1.99–2.46	8.00	3	7.29	1.94	1.76–2.16
**Rounded incisor embrasures**	6.00	3	6.07	2.34	2.12–2.62	7.00	3	6.29	2.18	1.98–2.43
**Incisal step of 1 mm**	8.00	2	7.83	1.87	1.69–2.09	8.00	2	7.74	1.67	1.52–1.86
**Incisal step of 0 mm**	7.00	3	7.25	2.13	1.93–2.38	8.00	4	7.31	1.80	1.64–2.01
**Diastema mediana of 0.5 mm**	6.00	3	5.57	2.09	1.89–2.33	6.00	3	5.56	2.06	1.87–2.29
**Diastema mediana of 1 mm**	6.00	3	5.39	2.15	1.95–2.40	6.00	3	5.45	1.99	1.81–2.22

*n*: number of participants.

**Table 3:** The differences between the experimental and control groups by individual occupations in the first testing.

	Experimental vs. Control Group									
Variable (*n* Experimental vs. *n* Control Group)	Laypeople (*n* = 84 vs. *n* = 86)		Students from 1st to 3rd Year (*n* = 26 vs. *n* = 12)		Students from 4th to 6th Year (*n* = 27 vs. *n* = 14)		Dentists (*n* = 28 vs. *n* = 42)		Dental Specialists (*n* = 9 vs. *n* = 32)	
	U	*p*	U	*p*	U	*p*	U	*p*	U	*p*
**Self-perception of smile**	3298.5	0.32	117.5	0.22	172	0.64	580	0.93	123	0.51
**Semi-rounded incisor embrasures**	3337.5	0.39	124.5	0.32	133.5	0.12	569.5	0.83	122	0.49
**Squared incisor embrasures**	3359	0.42	94.5	0.05	162	0.46	566.5	0.8	122	0.49
**Rounded incisor embrasures**	3286.5	0.31	130.5	0.43	163	0.48	567	0.8	87	0.07
**Incisal step of 1 mm**	3604	0.98	97.5	0.06	179	0.79	507.5	0.31	137	0.83
**Incisal step of 0 mm**	3441	0.59	139.5	0.61	169	0.59	588	1	107	0.23
**Diastema mediana of 0.5 mm**	3498	0.72	139.5	0.61	170.5	0.62	446.5	0.09	116.5	0.39
**Diastema mediana of 1 mm**	3226.5	0.23	145.5	0.75	174.5	0.69	467	0.14	107.5	0.25

*n*: number of participants.

**Table 4:** Mean scores, standard deviations, medians, and confidence intervals for each individual anomaly in the experimental and control groups in the second testing.

	Experimental Group (*n* = 174)					Control Group (*n* = 186)				
	Median	IQR	Mean	SD	(95% CI)	Median	IQR	Mean	SD	(95% CI)
**Self-perception of smile**	8.00	2	7.75	1.54	1.39–1.71	8.00	6	7.49	1.71	1.54–1.89
**Semi-rounded incisor embrasures**	7.00	2	7.09	1.99	1.80–2.22	8.00	7	7.50	1.72	1.55–1.91
**Squared incisor embrasures**	7.00	3	7.06	2.17	1.96–2.42	8.00	6	7.44	1.97	1.78–2.19
**Rounded incisor embrasures**	6.00	4	6.03	2.24	2.02–2.49	7.00	5	6.48	2.15	1.94–2.39
**Incisal step of 1 mm**	8.00	2	7.66	1.73	1.56–1.93	8.00	7	7.56	1.72	1.55–1.91
**Incisal step of 0 mm**	8.00	3	7.26	2.00	1.81–2.24	8.00	6	7.46	1.73	1.57–1.92
**Diastema mediana of 0.5 mm**	5.00	3	5.30	2.03	1.83–2.27	6.00	4	5.67	1.87	1.70–2.08
**Diastema mediana of 1 mm**	5.00	3	5.22	2.12	1.91–2.36	6.00	4	5.48	1.87	1.69–2.08

*n*: number of participants.

**Table 5:** The differences between the experimental and control groups by individual occupations in the second testing.

	Experimental vs. Control Group									
Variable (*n* Experimental vs. *n* Control Group)	Laypeople (*n* = 84 vs. *n* = 86)		Students from 1st to 3rd Year (*n* = 26 vs. *n* = 12)		Students from 4th to 6th Year (*n* = 27 vs. *n* = 14)		Dentists (*n* = 28 vs. *n* = 42)		Dental Specialists (*n* = 9 vs. *n* = 32)	
	U	*p*	U	*p*	U	*p*	U	*p*	U	*p*
**Self-perception of smile**	2924.5	0.03 *	132	0.46	186	0.95	515.5	0.39	138	0.86
**Semi-rounded incisor embrasures**	3305	0.34	145.5	0.75	124.5	0.08	578	0.91	130	0.67
**Squared incisor embrasures**	2933.5	0.03 *	130.5	0.43	177.5	0.76	501.5	0.3	88.5	0.08
**Rounded incisor embrasures**	3060.5	0.09	152.5	0.92	188.5	1	533.5	0.52	84.5	0.06
**Incisal step of 1 mm**	3546	0.84	113.5	0.19	182.5	0.87	457.5	0.12	120.5	0.46
**Incisal step of 0 mm**	3464.5	0.65	109.5	0.15	139	0.17	530.5	0.49	125	0.56
**Diastema mediana of 0.5 mm**	3257	0.27	149.5	0.85	154	0.34	546.5	0.62	121	0.47
**Diastema mediana of 1 mm**	3333	0.39	130	0.42	175.5	0.72	530.5	0.49	100.5	0.17

*n*: number of participants, * *p* < 0.05.

## Data Availability

All data used and/or analyzed during the current study are available from the corresponding authors upon reasonable request.
